# Aztreonam plus ceftazidime-avibactam for post-neurosurgical meningitis due to *Stenotrophomonas maltophilia*

**DOI:** 10.1128/aac.00172-25

**Published:** 2025-06-02

**Authors:** Jonathan Wong-So, Sophie Magréault, Étienne Carbonnelle, Vincent Jullien, Maxime Desgrouas, Thierry Boulain, François Barbier

**Affiliations:** 1 Service de Médecine Intensive Réanimation, Centre Hospitalier Universitaire d'Orleans 52817, Orléans, France; 2 Laboratoire de Pharmacologie, Hôpital Jean Verdier, Assistance Publique-Hôpitaux de Paris 26930, Paris, France; 3 Équipe IAME, INSERM, Université Sorbonne Paris Nord 167549, Bobigny, France; 4 Laboratoire de Microbiologie, Centre Hospitalier Universitaire d'Orleans 52817, Orléans, France; Houston Methodist Hospital and Weill Cornell Medical College, Houston, Texas, USA

**Keywords:** *Stenotrophomonas maltophilia*, aztreonam, ceftazidime-avibactam, central nervous system infections, therapeutic drug monitoring

## Abstract

Clinical evidence is lacking regarding the efficacy of aztreonam-avibactam in severe infections involving *Stenotrophomonas maltophilia*. We report a case of post-neurosurgical meningitis due to *S. maltophilia* (minimal inhibitory concentration of aztreonam plus avibactam, 4/4 µg/mL) clinically and microbiologically cured with a combination of aztreonam and ceftazidime-avibactam (2 g and 2 g/500 mg q8h, 3 h simultaneous infusions). Therapeutic drug monitoring showed adequate concentrations of aztreonam and avibactam in the cerebrospinal fluid at each time point over the 14-day treatment duration.

## INTRODUCTION

Healthcare-associated infections involving *Stenotrophomonas maltophilia* are challenging to manage owing to the paucity of available options for antimicrobial therapy. The species shows intrinsic resistance to broad-spectrum cephalosporins, carbapenems, and aztreonam due to the expression of two chromosomally encoded β-lactamases, namely, an L1 metallo-β-lactamase and an L2 inducible cephalosporinase (1). Aztreonam resists hydrolysis by L1 but is inactivated by L2, with the latter enzyme being potently inhibited by avibactam. Therefore, the combination of aztreonam and avibactam is active *in vitro* against more than 90% of the clinical isolates of *S. maltophilia*, including those with acquired resistance to trimethoprim-sulfamethoxazole and/or levofloxacin (2, 3). On the basis of these features, aztreonam-avibactam or, when locally unavailable, a combination of aztreonam and ceftazidime-avibactam are the preferred options for the treatment of *S. maltophilia* infections in the 2024 Infectious Diseases Society of America guidelines (4). However, clinical evidence on the efficacy of aztreonam-avibactam (combined or not with ceftazidime) is currently lacking in this indication, notably for central nervous system infections. Here, we report a case of post-neurosurgical meningitis due to *S. maltophilia* clinically and microbiologically cured with the association of aztreonam and ceftazidime-avibactam, with therapeutic drug monitoring (TDM) in the cerebrospinal fluid (CSF).

## CLINICAL CASE

A 59-year-old male patient with a history of hypertension and atrial fibrillation was admitted to the intensive care unit (ICU) for coma resulting from posterior fossa hemorrhage with secondary hydrocephalus in the context of warfarin overdose. An emergency neurosurgical intervention was performed for hematoma evacuation, decompressive posterior craniectomy, and insertion of an external ventricular drain. Intracranial hypertension persisted for several days, requiring extended sedation and ventricular drainage, with the latter being finally removed during the second week of the ICU stay. The patient was successively treated with intravenous ceftriaxone, cefepime, and amoxicillin for ventilator-associated pneumonia due to *Staphylococcus aureus* and *Hafnia alvei* and primary bloodstream infection due to *Enterococcus faecalis*. On the fourth week, dehiscence of the craniectomy wound with CSF leakage was noticed and managed with surgical fistula closure and insertion of a lumbar drain to lower the CSF pressure. The CSF samples collected during lumbar drainage (Day 2) and through the drain the day after showed neutrophilic pleocytosis and biochemical features compatible with post-neurosurgical meningitis (Table S1 in the electronic supplement). Cultures of these CSF samples were both positive for *S. maltophilia* (species identification through matrix-assisted laser desorption/ionization time-of-flight mass spectrometry, Bruker, Germany). Routine antimicrobial susceptibility testing was performed following the Canadian Society for Microbiology and Infection Control/European Committee on Antimicrobial Susceptibility Testing (EUCAST) guidelines (5). The *S. maltophilia* isolate was susceptible to minocycline (not available in France) and levofloxacin but showed resistance to trimethoprim-sulfamethoxazole.

## MULTIPLE-CHOICE QUESTION

What antimicrobial regimen would you have considered to treat this patient?

1. Levofloxacin monotherapy
2. Levofloxacin plus colistin
3. Levofloxacin plus high-dose tigecycline
4. Aztreonam plus avibactam (combined or not with ceftazidime)
5. Cefiderocol monotherapy

## TREATMENT STRATEGY AND PATIENT OUTCOMES

The administration of levofloxacin alone was not retained due to conflicting evidence regarding the efficacy of single-drug therapy in severe infections caused by *S. maltophilia* (4). A levofloxacin plus cefiderocol combination could have been considered (4); however, available data did not plead for the use of cefiderocol as part of the first-line regimen in this indication (6). Tigecycline, a potential last-resort option for *S. maltophilia* infections, and colistin, which is poorly active against this species, were not tested *in vitro* (4).

The minimal inhibitory concentrations (MICs) of aztreonam, ceftazidime, ceftazidime-avibactam, and aztreonam-avibactam (fixed avibactam concentration, 4 µg/mL) measured using broth microdilution assays (7) were >256, >256, 32/4, and 4/4 µg/mL, respectively. There is currently no established MIC cut-off to define aztreonam-avibactam susceptibility in *S. maltophilia*; however, a value of 8 µg/mL—i.e., the Clinical and Laboratory Standards Institute (CLSI) breakpoint for *Pseudomonas aeruginosa*—has been used in most bodies of research in the field (3, 7).

Aztreonam-avibactam was not yet commercially available in our hospital at the time of patient admission. Therefore, a combination of ceftazidime-avibactam (2 g/500 mg q8h) and aztreonam (2 g q8h) was initiated (Day 0). Both antimicrobials were administered simultaneously by 3 h infusions—in accordance with the Infectious Diseases Society of America guidelines (4)—for a total duration of 14 days. On Day 0, the patient’s weight was 56 kg (without fluid overload); albuminemia was 24 g/L; and the estimated glomerular filtration rate was 78 mL/min/1.73 m^2^ (Chronic Kidney Disease Epidemiology Collaboration formula)—the renal function remained stable over the 14 days of therapy.

The peak and trough total concentrations of aztreonam, ceftazidime, and avibactam were measured in the plasma and/or the CSF (sampling through the lumbar drain) as part of routine TDM at days 0, 1, 2, 3, 4, 7, 12, and 13 using liquid chromatography/tandem mass spectrometry (8, 9). The CSF-to-plasma concentration ratios were calculated when coupled measures were available on a given day.

The mean (± standard deviation) plasma peak and trough concentrations of aztreonam (106.8 ± 15.2 and 51.8 ± 5.8 µg/mL), ceftazidime (119.9 ± 12.2 and 87.3 ± 10.3 µg/mL), and avibactam (15.3 ± 5.3 and 6.8 ± 0.9 µg/mL) were in the upper range of those previously reported in critically ill individuals with a similar dosing regimen (Table 1; Fig. S1 in the electronic supplement) (10–15). This could ensue from the low patient’s weight, the absence of fluid overload (i.e., no increase in the distribution volume of the drugs), the normal renal function (no augmented renal clearance), and, for aztreonam, the dosing of total rather than unbound fractions in the context of relative hypoalbuminemia—indeed, protein binding is ~50% for aztreonam (12) and <10% for ceftazidime and avibactam (1). The inter-study variability in dosing methods might also have contributed to this observation; yet, liquid chromatography/tandem mass spectrometry has shown high sensitivity and accuracy for β-lactam TDM (8). Plasma concentrations of the three drugs remained stable throughout the duration of antimicrobial therapy.

**TABLE 1:** Results of the therapeutic drug monitoring of aztreonam, ceftazidime, and avibactam in plasma and cerebrospinal fluid samples^*a*^^,^^*b*^

Day of sampling	Peak plasma concentrations, µg/mL			Trough plasma concentrations, µg/mL			Peak CSF concentrations, µg/mL			Trough CSF concentrations, µg/mL		
	AZT	CAZ	AVI	AZT	CAZ	AVI	AZT	CAZ	AVI	AZT	CAZ	AVI
Day 0	-	-	-	45.3	85.4	9.5	-	-	-	-	-	-
Day 1	105.0	133.0	14.0	47.7	80.8	5.7	13.5	42.9	1.4	20.6	56.0	1.6
Day 2	108.0	130.0	12.4	51.8	80.0	5.5	19.8	56.7	1.4	20.0	52.9	1.7
Day 3	110.0	117.0	14.3	62.8	105.0	7.8	28.2	64.3	1.8	18.9	71.9	2.0
Day 4	105.0	115.0	13.1	50.0	83.9	5.9	19.7	53.7	1.2	20.4	56.8	1.5
Day 7	130.3	124.5	25.9	53.4	88.6	6.2	-	-	-	9.0	28.6	1.0
Day 12	-	-	-	-	-	-	-	-	-	10.1	30.3	1.2
Day 13	82.5	99.6	12.2	-	-	-	7.2	24.8	0.8	-	-	-
Mean (SD)	106.8 (15.2)	119.9 (12.2)	15.3 (5.3)	51.8 (5.8)	87.3 (10.3)	6.8 (0.9)	17.7 (7.9)	48.5 (15.3)	1.3 (0.4)	16.5 (5.4)	49.4 (16.8)	1.5 (0.4)

^*a*^ - indicates that concentrations were not measured at the corresponding time points.

^*b*^ CSF, cerebrospinal fluid; AZT, aztreonam; CAZ, ceftazidime; AVI, avibactam; SD, standard deviation.

**Fig 1:**
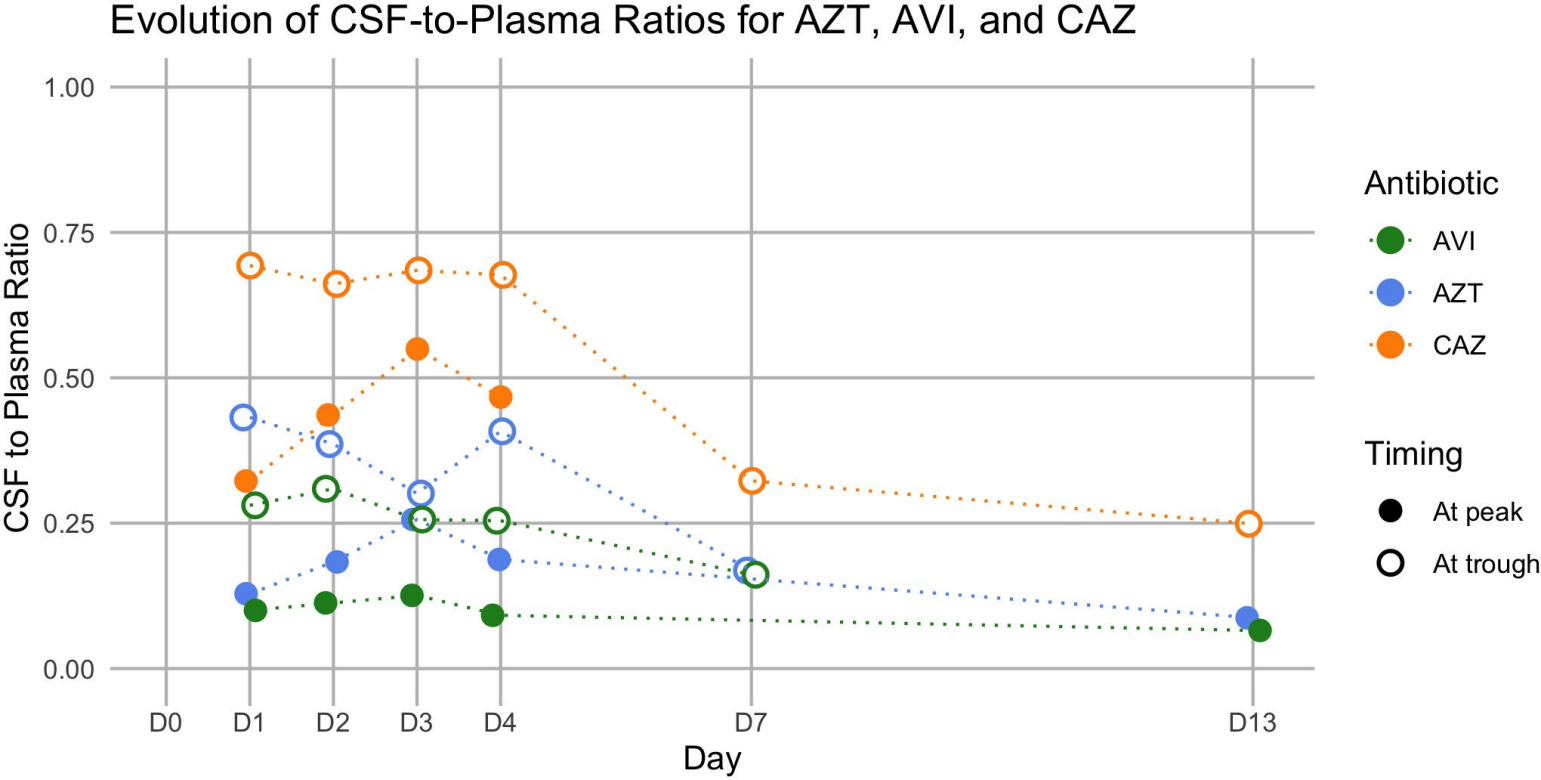
Temporal evolution of the cerebrospinal fluid-to-plasma concentration ratios of aztreonam, ceftazidime, and avibactam. CSF, cerebrospinal fluid; AZT, aztreonam; CAZ, ceftazidime; AVI, avibactam.

The mean CSF peak and trough concentrations of aztreonam (17.7 ± 7.9 and 16.5 ± 5.4 µg/mL, respectively) and ceftazidime (48.5 ± 15.3 and 49.4 ± 16.8 µg/mL, respectively) were higher than those usually observed in patients with meningitis (16–23) possibly due to dosing performed early after meningitis onset and the use of extended rather than intermittent infusion schemes, though this latter point remains debated (24, 25). Trough concentrations were equal to or higher than the peak concentrations for both antimicrobials (Table 1; Fig. S1), as were the CSF-to-plasma concentration ratios (Fig. 1), indicating delayed CSF diffusion and slower CSF than plasma clearance (26). Also, the CSF-to-plasma concentration ratios decreased over time (Fig. 1), which may result from reduced CSF diffusion as meningeal inflammation resolved (16, 21). The estimated area under the receiving curves (AUC) of plasma and the CSF concentrations are exposed in Table S2, with the corresponding CSF-to-plasma AUC ratios. Importantly, the peak and trough concentrations of aztreonam in the CSF were >4 µg/mL at each time point, that is above the MIC of the *S. maltophilia* isolate (when combined with avibactam) for the entire duration of inter-dose intervals (*ƒ*T > MIC = 100%). Likewise, the CSF peak and trough concentrations of ceftazidime over the first week of therapy were consistently >32 µg/mL, with this value corresponding to the measured MIC of ceftazidime when combined with avibactam.

The mean CSF peak and trough concentrations of avibactam (1.3 ± 0.4 and 1.5 ± 0.4 µg/mL, respectively) were consistent with available data. In four adult patients receiving ceftazidime-avibactam (2 g/500 mg q8h, 2 to 3 h infusions) for meningitis due to multidrug-resistant *P. aeruginosa* or Enterobacterales, peak or early post-infusion concentrations of avibactam in the CSF ranged from 0.9 to 4.2 µg/mL, while inter-dose or trough concentrations ranged from 1.4 to 2.4 µg/mL (18, 19). Similar concentrations were reported in a pediatric patient with post-neurosurgical meningitis due to extended-spectrum β-lactamase-producing *Escherichia coli* (27). As for aztreonam and ceftazidime, trough concentrations were equal to or higher than peak concentrations, with a higher CSF-to-plasma concentration ratio at trough, suggesting delayed CSF diffusion. At each time point, the peak and trough concentrations of avibactam were >1 µg/mL, a value that appears sufficient for inhibition of most β-lactamases in Enterobacterales and *P. aeruginosa* (28–31). To the best of our knowledge, the critical concentration (C*_T_*) of avibactam for inhibition of the L2 cephalosporinase of *S. maltophilia* has not been determined yet. Further investigations are warranted to appraise whether efficient killing of *S. maltophilia* may be obtained with avibactam concentrations below 4 µg/mL, the current CLSI and EUCAST fixed values for susceptibility testing when combined with aztreonam.

Sequential CSF analyses showed a steady decrease in leukocytosis, proteinorachia, and lactate level (Table S1). Cultures of CSF samples collected at days 2, 4, 7, and 13 were negative for *S. maltophilia*, indicating prompt microbiological eradication with aztreonam-avibactam. We cannot exclude, however, that the co-administration of ceftazidime has contributed to this favorable outcome. To the best of our knowledge, this is the first reported microbiological cure of a central nervous system infection due to *S. maltophilia* with ceftazidime-avibactam plus aztreonam; nevertheless, a successful eradication of metallo-β-lactamase-producing gram-negative bacteria in the CSF with this combination has been previously observed in a patient with meningitis due to a New Delhi metallo-β-lactamase-producing *Klebsiella pneumoniae* strain (32).

The dosing scheme remained unchanged over the 14 days of antimicrobial therapy owing to microbiological success (despite avibactam CSF concentrations below 4 µm/mL) and gradual improvement in neurological status, without argument for β-lactam-induced encephalopathy. The patient was weaned from mechanical ventilation during the fifth week of the ICU stay, subsequently transferred to a medicine ward, and discharged home 76 days after hospital admission without significant residual disability (modified Rankin scale equal to 1).

The patient provided written informed consent for the publication of anonymized health data in accordance with institutional rules.

## CONCLUSION

This case suggests that a combination of ceftazidime-avibactam and aztreonam may be an efficient option for healthcare-associated meningitis due to *S. maltophilia* and, possibly, other metallo-β-lactamase-producing gram-negative pathogens. Cohort studies are necessary to confirm these findings and assess whether they are reproducible with aztreonam-avibactam alone since this drug is now Food and Drug Administration-approved and will be shortly available for clinical use.

## SUPPLEMENTAL MATERIAL

Supplemental material aac.00172-25-s0001.docx Tables S1 and S2; Fig. S1.

ASM does not own the copyrights to Supplemental Material that may be linked to, or accessed through, an article. The authors have granted ASM a non-exclusive, world-wide license to publish the Supplemental Material files. Please contact the corresponding author directly for reuse.
