# Supplementary material for: Aztreonam plus ceftazidime-avibactam for post-neurosurgical meningitis due to Stenotrophomonas maltophilia
Source: Antimicrob Agents Chemother. 2025 Jun 2;69(8):e00172-25. doi: 10.1128/aac.00172-25 (PMC12327007; doi:10.1128/aac.00172-25)
Supplement: Supplemental material — Tables S1 and S2; Fig. S1. [file aac.00172-25-s0001.docx]

**Electronic supplementary material**

**Aztreonam plus ceftazidime-avibactam for post-neurosurgical meningitis due to *Stenotrophomonas maltophilia***

Jonathan Wong-So,^1^ MD; Sophie Magréault,^2,3^ PharmD, PhD; Étienne Carbonnelle,^4^ MD, PhD; Vincent Jullien,^2,3^ PharmD, PhD; Maxime Desgrouas,^1^ MD, MSc ; Thierry Boulain,^1^ MD; François Barbier,^1^ MD, PhD

**Author affiliations:**

^1^ Service de Médecine Intensive Réanimation, Centre Hospitalier Universitaire d’Orléans, Orléans, France ; ^2^ Laboratoire de Pharmacologie, Hôpital Jean Verdier, Assistance Publique-Hôpitaux de Paris, Bondy, France ; ^3^ Équipe IAME, INSERM, Université Sorbonne Paris Nord, Bobigny, France ; ^4^ Laboratoire de Microbiologie, Centre Hospitalier Universitaire d’Orléans, Orléans, France

**Table e1.** Results of cerebrospinal fluid analyses

| Day | Leukocytes, cells per mm^3^ | Neutrophils, % | Lymphocytes, % | Erythrocytes, cells per mm^3^ | Protides, g/L | Glucose, mmol/L | Lactate, mmol/L | Culture |
| --- | --- | --- | --- | --- | --- | --- | --- | --- |
| Day -2 | 180 | 76 | 4 | 6300 | NA | NA | NA | Positive for *S. maltophilia* |
| Day -1 | 850 | 87 | 4 | 3400 | 2.6 | 2.2 | 7.4 | Positive for *S. maltophilia* |
| Day 0 | 850 | 87 | 4 | 3400 | 1.7 | 2.0 | 7.1 | Positive for *S. maltophilia* |
| Day 2 | 160 | 70 | 30 | 220 | NA | NA | NA | No growth |
| Day 4 | 100 | 12 | 74 | 170 | NA | NA | NA | No growth |
| Day 7 | 50 | 20 | 80 | 240 | 1.1 | 1.3 | 6.8 | No growth |
| Day 13 | 6 | - | - | 600 | 0.78 | 1.8 | 5.5 | No growth |

*Table e1 footnote*

Day 0 corresponds to the day of initiation of antimicrobial therapy combining aztreonam and ceftazidime-avibactam.

Cerebrospinal fluid cultures were performed on solid media and liquid broth according to routine procedures.

NA, non-available

**Table e2.** Aztreonam, ceftazidime and avibactam concentrations in the plasma and cerebrospinal fluid: estimated AUC

| Day of sampling | AUC of aztreonam concentrations | | |  | AUC of ceftazidime concentrations | | |  | AUC of avibactam concentrations | | |  |
| --- | --- | --- | --- | --- | --- | --- | --- | --- | --- | --- | --- | --- |
|  | Plasma | CSF | CSF-to-plasma ratio |  | Plasma | CSF | CSF-to-plasma ratio |  | Plasma | CSF | CSF-to-plasma ratio |  |
| Day 1 | 381,7 | 85,2 | 0,22 |  | 534,5 | 247,2 | 0,46 |  | 49,2 | 7,5 | 0,15 |  |
| Day 2 | 399,5 | 99,5 | 0,25 |  | 525 | 274,0 | 0,52 |  | 44,7 | 7,7 | 0,17 |  |
| Day 3 | 432,0 | 117,7 | 0,27 |  | 555 | 340,5 | 0,61 |  | 55,2 | 9,5 | 0,17 |  |
| Day 4 | 387,5 | 100,2 | 0,26 |  | 497,25 | 276,2 | 0,56 |  | 47,5 | 6,7 | 0,14 |  |

*Table e2 footnote*

AUC are exposed as µg/mL x hours.

AUC were estimated according to a non-compartimental approach as (peak concentration + trough concentration)/2 x 5 hours (5 hours being the time elapsed between peak concentration measurements at the end of extended 3-hour infusion, and trough concentration measurements, immediately before the administration of the following dose).

Please note that CSF-to-plasma AUC ratios could not be estimated at other time points as coupled measures were not available.

AUC, area under the receiver operating curve; CSF, cerebrospinal fluid

**Figure e1.** Results of therapeutic drug monitoring of aztreonam, ceftazidime and avibactam in plasma and cerebrospinal fluid samples


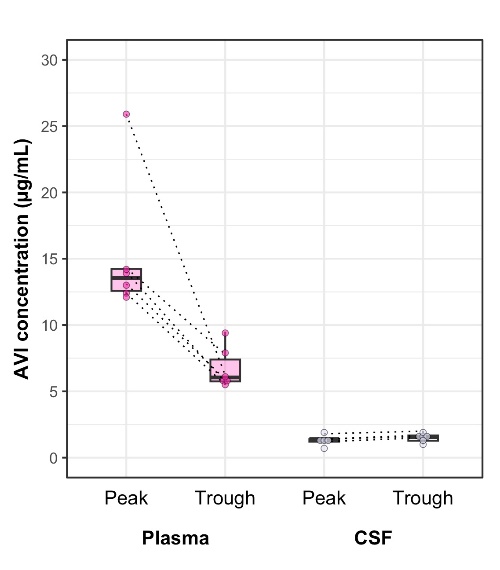

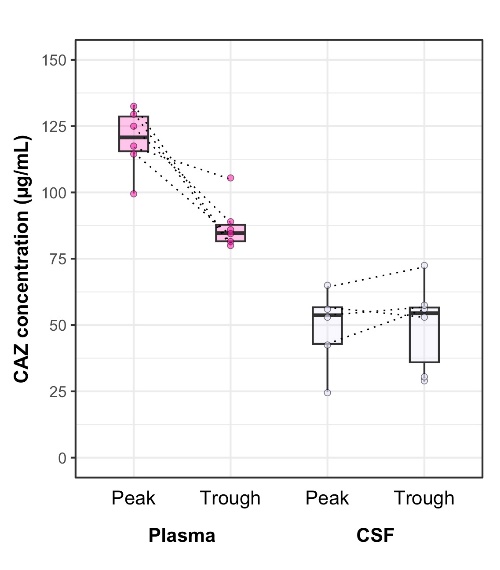

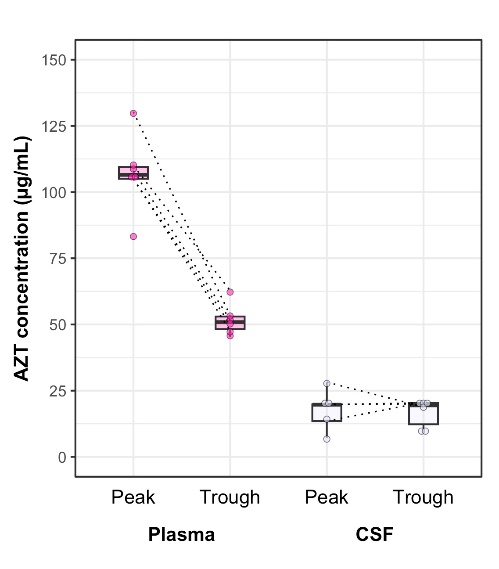


*Figure e1 footnote*

CSF, cerebrospinal fluid; AZT, aztreonam; CAZ, ceftazidime; AVI, avibactam

Boxplots indicate median values and corresponding interquartile ranges.
